# XHM: A system for detection of potential cross hybridizations in DNA microarrays

**DOI:** 10.1186/1471-2105-5-117

**Published:** 2004-08-27

**Authors:** Kristian Flikka, Fekadu Yadetie, Astrid Laegreid, Inge Jonassen

**Affiliations:** 1 Computational Biology Unit, Bergen Center for Computational Science, UNIFOB/UiB, Thormoehlensgt.55, N-5008 Bergen, Norway; 2 Department of Cancer Research and Molecular Medicine, Norwegian University of Science and Technology, NO-7489 Trondheim, Norway; 3 Sars International Centre for Marine Molecular Biology, Bergen High Technology Centre, Thormoehlensgt. 55, N-5008 Bergen, Norway; 4 Department of Informatics, University of Bergen, PB. 7800, N-5020 Bergen, Norway

## Abstract

**Background:**

Microarrays have emerged as the preferred platform for high throughput gene expression analysis. Cross-hybridization among genes with high sequence similarities can be a source of error reducing the reliability of DNA microarray results.

**Results:**

We have developed a tool called XHM (cross hybridization on microarrays) for assessment of the reliability of hybridization signals by detecting potential cross-hybridizations on DNA microarrays. This is done by comparing the sequences of the probes against an extensive database representing the transcriptome of the organism in question. XHM is available online at .

**Conclusions:**

Using XHM with its user-adjustable parameters will enable scientists to check their lists of differentially expressed genes from microarray experiments for potential cross-hybridizations. This provides information that may be useful in the validation of the microarray results.

## Background

The development of DNA microarrays has revolutionized high throughput gene expression analysis. The two main platforms are: cDNA microarrays, where PCR products of individual cDNA fragments are immobilized on glass slides [1], and oligonucleotide microarrays, where oligonucleotides *in situ *synthesized or spotted on glass slides are used [2,3]. In a typical cDNA microarray experiment, total cellular RNA from two sources, a reference (or control) and an experimental sample are converted to cDNA by reverse transcription, labeled with two different fluorescent colors, and hybridized to an array of cDNA probes.

One of the major concerns of cDNA microarrays is cross-hybridization of the labeled RNA (or cDNA) to non-target homologous probe sequences on the array [4,5]. Cross-hybridizations that may arise due to poly(A)-tail of mRNA or repetitive elements may be reduced or eliminated using hybridization blocking reagents like poly(A) oligonucleotides [6] or Cot1 DNA. Another major source of cross-hybridization may come from conserved sequences shared by two or more cDNAs, such as gene family members [4,7]. Analysis of sequenced multicellular eukaryotic genomes suggests that a large percentage of genes belong to gene families, some of which have high sequence similarities [8]. A recent study reported that approximately 17% of human transcripts in the UniGene database contain perfect match repeats of 20 bp minimum lengths [9]. From this study, however, the frequency of long, non-perfect match sequences such as those shared among paralogs, was not clear.

Some studies have suggested that cross-hybridization can be a source of errors in cDNA microarray experiments [3,4,7,9,10]. In general, these studies indicate that cDNAs with nucleotide sequence identities higher than 70–80% over a certain length show significant levels of cross-hybridization. Analysis of chemokines, cytochrome P450 isozymes, G proteins and protease homologous gene families showed that cross-hybridization signals of 0.6–12% and 26–57% could arise from shared nucleotide identities of 55–80% and >80%, respectively [4]. Using synthetic 50 mer oligonucleotides, it was also shown that non-target sequences with >75–80% identity to 50 mer probes in microarrays could result in cross-hybridization [3]. In addition, cross-hybridization was also observed if a non-target sequence included stretches of ≥ 15 continuous bases identical to a 50 mer probe sequence [3]. Cross-hybridization is thought to contribute to discrepancies of results observed between oligonucleotide arrays and cDNA arrays [11].

Cross-hybridization may occur for both oligonucleotide and cDNA microarrays. An advantage with oligonucleotide arrays is that the complete sequences of the probes are known. Because cDNA arrays are constructed from PCR products of cDNA clones sequenced from 3'- or 5'-ends, the complete sequences of the spotted probes may not be available.

We have not been able to identify programs or web servers capable of doing flexible cross-hybridization analysis. Programs with related functionality have been described, including ProbeWiz [12], OligoWiz [13] and PROBEmer [14], but these are for designing specific probes by avoiding regions of the cDNAs where there may be a cross-hybridization problem. For the assessment of the cross-hybridization potential in DNA microarray analysis, a more specific tool is required.

In the present study we limit ourselves to the following problem: Given a probe (or a set of probes), identify the targets to which it can hybridize given user-defined criteria.

The exact criteria to be used to decide whether two genes with high sequence similarity can cross-hybridize will depend on a number of factors, including the set of probes used and the experimental protocols – in particular hybridization and washing stringencies. We propose to use generic forms of criteria allowing the user to choose parameters in order to define the criteria for cross-hybridization.

We investigate whether BLAST can be used to detect targets satisfying a set of criteria for cross-hybridization, based on previous experimental findings [3,4], and assess different (transcript) databases in order to evaluate their suitability for our purpose. A web tool is developed that allows the user to query precomputed results from several probe sets, or to analyze own probes with respect to a database representing transcripts in the organism where the microarray experiments are performed. The tool is applicable to analysis of both oligonucleotide and cDNA probes.

Below we describe the developed XHM (cross hybridization on microarrays) system. We also include analysis performed in order to identify appropriate analysis methods and databases. Furthermore we describe the web server for XHM. In the final section we report some example results using our system on three cDNA probe sets for human, mouse and rat, and one oligonucleotide probe set for mouse.

## Implementation

The XHM system queries the given nucleotide sequences (for example, a list of differentially regulated genes from DNA microarray experiments) against a database representing all possible targets (transcripts) in the organism being studied, and produces a list of all targets that can hybridize to each input sequence under the defined criteria.

In this section we evaluate databases for use in the XHM system and we evaluate whether BLAST can be used to identify potential cross-hybridizing genes.

### Definitions

For the calculation of melting temperature, the following formula is used [15]:

*Tm = *81.5 + 16.6 * *log *[*Na*^+^] + 41 * (*numG + numC*)/*n *- 500/*n*

where [*Na*^+^] is the *Na*^+^concentration, *numG *and *numC *is the number of Gs and Cs in the given alignment, and *n *is the total number of nucleotides aligned.

We differentiate between two kinds of sequence similarity cutoffs for cross-hybridization, based on the situations described for oligonucleotide microarrays [3]:

• *Type A *similarities for sequence segments of a certain minimum length with a defined minimum percentage identity (long alignments with mismatch).

• *Type B *similarities for identical segments of some minimum length (short perfect match).

To perform the searches in the different databases, NCBI BLAST version 2.2.1 (Mon Jul 9 14:02:00 EDT 2001) [16] was used. The default settings of *blastn *are used unless specified otherwise. This includes using the DUST filter for masking lowcomplexity regions.

### Databases and sequences

In this study we used three cDNA clone sets (corresponding to three cDNA sets spotted on microarrays). The 40 k *Homo sapiens *(Hs) and the 14 k *Rattus norvegicus *(Rn) clone sets are I.M.A.G.E. Consortium [LLNL] cDNA clones [17,18] obtained from Research Genetics (Huntsville, AL). The 15 k *Mus musculus *(Mm) clone set is from NIA [19]. In addition, oligonucleotide sequences (Mouse Genome Array Ready Oligonucleotide Set) from QIAGEN Operon [20] have been used to test the application. The details of the databases used to search for possible cross-hybridizations are as follows:

• RefSeq Version 2 – RefSeq mRNA collection [21]. Number of sequences: 25377/21200/21724 (mouse/rat/human).

• UniGene – UniGene Unique, build 129/123/164 (mouse/rat/human) [22]. Number of sequences: 87495/50137/118326 (mouse/rat/human).

• TIGR gene index [23] – Only tentative consensus sequences (TCs), version 020403/042503/020503 (mouse/rat/human). Number of sequences 58129/51330/187287 (mouse/rat/human).

• BeGIn – Bergen Gene Index version 1.0. Number of sequences: 100654/49285 (mouse/rat).

BeGIn is an alternative database we have developed (using tools described in [24]), by producing consensus sequences from each cluster in UniGene (build 129/123 for mouse/rat).

In order to achieve high specificity and sensitivity, we favored the databases where the highest number of probes were represented (completeness) and multiple matches per probe were minimized (minimum redundancy). Our criteria for choosing an appropriate database for a given organism was based on the results obtained for our probe sets. The presence of alternative splice forms in either the probe or the target set may complicate analysis. We do not consider this explicitly in the current study.

### Overview of the XHM system

**Figure 1:**
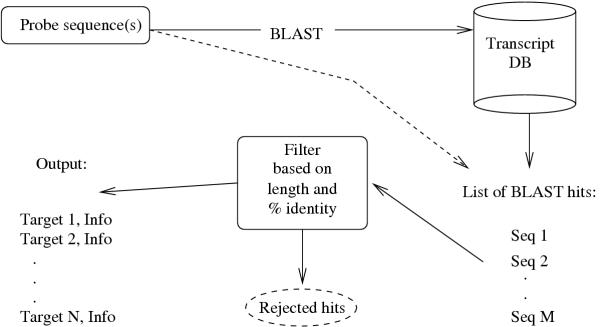
**Main components of the XHM system. **One or several query sequences (or identifiers) are entered, a BLAST search is performed, the resulting hits are filtered based on length and similarity, and the results are displayed. The dashed arrow symbolizes the possibility to use a sequence id whose hits to the database have been stored.

The basis of the XHM system is a BLAST search (see Figure 1). The BLAST search provides one or several alignments between the probe sequence and each of the possible cross-hybridizing target sequences. The alignments are analyzed with respect to whether they fulfill the user-defined criteria for *type A *or *type B *matches. The XHM system presents to the user a list of matches satisfying the criteria, and in addition information on GC-content in similar regions, estimated melting temperature (Tm) and position on the sequence (proximity to 3' or 5' end). Although the estimated Tm may not be completely accurate under microarray hybridization conditions, particularly on solid surfaces, it can give a relative measure of the impact of the potential cross-hybridization detected. An expert user may use the information to assess the likelihood and importance of the cross-hybridization effect.

### Appropriateness of BLAST

Experiments were performed to assess whether BLAST is able to identify potentially cross-hybridizing targets. The analysis was done by generating simulated targets (*T*_1_...*T_n_*) for a number of probes (*P*_1_...*P_n_*). The simulated targets were ensured to satisfy the criteria described in [3] (50 bp sequences with at least 75% identity or 15 bp identical sequence). BLAST was then used to query the original probes against a large database containing the designed targets. It was then checked whether or not *T_i _*was contained in *P_i_*'s hitlist.

We found that *type A *matches can be missed if they do not contain any one stretch identical to the probe of length larger than BLAST's initial word length. Our experiments also showed that, depending on the probe type and the criteria used, we sometimes had to allow BLAST to generate very long lists of hits and using very high (permissive) cutoffs on E-value in order to cover the intended targets. Part of the reason for this is that BLAST is designed to identify homologous sequences and the sequences satisfying the cross-hybridization criteria need not necessarily receive significant scores or E-values. Our conclusion was that BLAST can be used, but to achieve optimal results, non-default parameters for initial word size and mismatch penalty should be applied. This was taken into consideration in the web-server.

### A web server

A web interface has been designed  with two entry points for accessing cross-hybridization information (see Figure 1). The user may either query a database of precomputed alignments, or compute new alignments using a real-time BLAST search. Both versions have parameters for the different thresholds and output alternatives. For ease of use, it is possible to run queries with no parameters explicitly specified.

When querying with a large number of clones against a large database, running BLAST may be time consuming. Therefore, we pre-run BLAST with different clone-sets against different databases, and store the results. In this way the user may experiment with different thresholds for a set of clones repeatedly, and get the results within seconds.

Nucleotide sequences or GenBank accession numbers may be used as input to the system when running the real-time BLAST searches. Searching using the precomputed BLAST alignments does not accept nucleotide sequences as input.

#### User-adjustable input parameters

Some of the parameters are shared among the two main versions of the XHM system. These include minimum length and minimum percentage similarity for *type A *hits (defaults are 75% over 50 bp) and minimum length for *type B *hits (default is 15 bp), based on the findings described in [3], Na^+ ^concentration (default is 0.1 M), and size of GC-clamp (default is 10). The Na^+ ^concentration is used to calculate melting temperature, and the size of the GC-clamp is used to plot the GC-content throughout the query sequence.

In addition, the real-time BLAST version also contains BLAST specific user-adjustable parameters. Main parameters include threshold on E-value (default is 10), number of alignments in the output from BLAST and whether DUST (low complexity) filter should be used. The user can adjust other BLAST specific parameters including gap opening and extension penalties, initial word size, mismatch penalty and whether or not to allow gapped alignments.

#### Output

**Figure 2:**
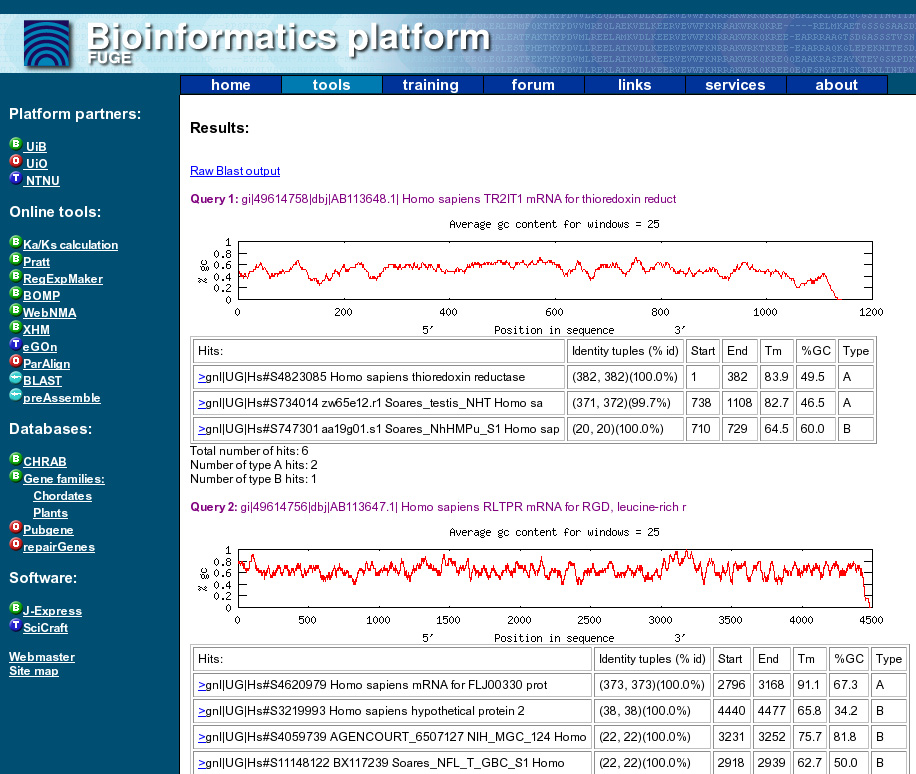
**Screenshot of the XHM web output. **The screenshot shows an example output from the XHM system.

The output from the XHM system consists of different parts (see Figure 2). If there are several input sequences (batch query), the results are given for each sequence individually. For each probe the system generates a plot of GC-content along the sequence. This GC-plot may help the user to identify areas in the sequence with high GC content in order to evaluate the importance of the potential cross-hybridization detected.

For each hit (possible hybridization), XHM presents the name and identifier of the hit, identity tuples (from BLAST – number of identical bases and number of bases in total in the BLAST alignment, or in a sub-alignment), start and end position on the probe, calculated Tm, percentage GC in alignment and type of hit (A or B).

## Results

### Choice of database

Experiments using the three cDNA sets and the oligonucleotide set as input to the XHM system yielded the results shown in Table 1. Using a 70% identity threshold for the *type A *similarities, in the rat clone set the Rat Gene Index from TIGR appears to be the best choice. For the mouse clone set it appears that the BeGIn database is the best choice, and for the human clone set it seems that RefSeq or UniGene Unique may be two good choices. These evaluations were based on completeness (high number of probes represented) and minimum redundancy of the database. We used only the tentative consensuses (TCs) of the Gene Indices databases from TIGR. Using the "full" versions decreased the number of probes having zero hits, but substantially increased the number of probes having two or more hits (results not shown).

### Application to microarray probes

As a practical experiment, we tested the XHM system on the cDNA probe sets for human, mouse and rat, and the mouse oligonucleotide set from QIAGEN. For the cDNA sets we used the following thresholds: 70% over 200 nucleotides for *type A *similarities, and 25 nucleotides for the *type B *similarities. For the oligonucleotides we used 70% identity over the whole oligonucleotide sequence (*type A*) and 20 nucleotides perfect identity (*type B*). Choosing the parameters is not trivial, and these thresholds, which are in the lower range of % similarities leading to cross-hybridizations, are meant as examples of a possible configuration. The XHM tool allows for full flexibility to experiment with the settings. The reason why we chose to consider a 200 nucleotides stretch for the cDNA probes is that sometimes these sequences contain errors, especially toward the ends.

Results are shown in Table 1. Using RefSeq, we observed that even though a substantial number of the 14 k rat cDNA probes (63.3%) had no *type A *hit, 809 (about 16.3% of the probes actually represented in RefSeq) had two or more hits, indicating potential cross-hybridization. In most cases, a single hit represents the probe sequence itself, and does not represent a potential cross-hybridization. Looking at a more complete database, the TIGR Rat Gene Index, only 700 (5.2%) probes had no hits, and 2712 (21.3% of the probes represented in the database) had two or more hits. A conclusion from this was that the number of rat probes having potential cross-hybridizing partners was between 16% and 21%.

For the mouse cDNA probes the cross-hybridization numbers appeared to be higher. Using RefSeq, the number of probes having two or more *type A *hits was 3101 or 34.2% of the probes represented in the database. RefSeq is relatively incomplete, so this number may be a conservative estimate on the cross-hybridization occurrence. For the BeGIn database only 725 probes had no hits (5.3%) and 5211 probes (about 40% of the probes in the database) had two or more hits.

Even though as many as 47.3% of the mouse probes were not found in the TIGR Gene Index, the proportion of probes found in the database that had two or more hits was 59%. This number is most likely an artifact caused by redundancy in the database.

The difference in number of possible hybridizations found in mouse versus rat is most likely due to the fact that more mouse sequences are available.

The human cDNA probes seems to have a lower cross-hybridization potential than the mouse probes. In UniGene Unique and RefSeq, looking only at the human probes that were found in the databases, just above 30% of them had two or more hits. The results using TIGR Human Gene Index suggested that almost 70% of the probes found in the database could cross-hybridize, but this is most likely an artifact caused by redundancy.

The mouse oligonucleotide set is clearly less prone to cross-hybridization. Using the UniGene Unique database, we observed that 14% of the probes found in the database had two or more hits, whereas the corresponding number for the 15 k mouse cDNA probe set using UniGene Unique was 45%.

## Discussion

A flexible tool for assessing the cross-hybridization potential of microarray probes has been developed and made available. Several transcriptome databases can be used for searching, and more may be added upon request. Using the XHM tool, analysis of three cDNA microarray probe sets and one oligonucleotide probe set revealed that a high proportion of the cDNA probes can potentially cross-hybridize with one or more other transcripts in the organism. As expected, compared to the cDNA probes, a smaller percentage of the mouse oligonucleotide probes showed a potential for cross-hybridization. This is because the oligonucleotide probe sequences are much shorter (69-mers), designed from specific regions of cDNAs to minimize cross-hybridizations. Despite increasing use of the more specific oligonucleotide arrays, cDNA sequences including full-length clone sets [25,26] are widely used in production of microarrays.

Depending on the completeness and redundancy levels of the transcriptome databases used, with the chosen cutoff for *type A *similarities (at least 70% identity), 15–45% of the cDNA probes showed hybridization with two or more apparently different transcripts (disregarding the results using the TIGR Gene Indices). This high percentage of potentially cross-hybridizing genes suggests that it is essential to carefully validate results from microarray experiments, particularly where cDNA clones are used to prepare arrays.

Although cross-hybridization is known as one of the main sources of errors of cDNA microarrays, the high proportions of cross-hybridizing genes detected in our test are likely to be overstated by the possible redundancies in the databases (not all entries represent unique genes). Also one may argue that the 70% identity threshold is somewhat low.

Whether two different genes that are candidates for cross-hybridization actually lead to erroneous results in hybridization experiments will depend on factors such as the level of sequence identity, the stringency of the hybridization and the relative abundances of the transcripts. For example, two potentially cross-hybridizing genes do not necessarily pose a problem unless both are expressed in the tissue or cell-line analyzed. Quick inspection of the hit-list produced by the XHM tool for a typical input of differentially regulated genes will help in identifying significant noise from cross-hybridizations. A main advantage of the XHM tool is that it allows the user to perform searches at various stringencies to detect potential cross-hybridizations. Candidate genes from microarray analysis that show potential cross-hybridizations using the database search may then be further checked using other methods such as RT-PCR and Northern blot.

## Conclusions

We have shown that a significant proportion of probes used in cDNA microarray analysis may show cross-hybridization with non-target sequences. We have developed a flexible tool, XHM, suitable for detecting potential cross-hybridization artifacts during microarray data analysis. The tool may also be used to select specific probes for preparation of microarrays.

## Availability

The XHM system is freely available at . Program code for academic use can be supplied upon request to the author.

## Author's contributions

KF implemented the system and made a draft of the manuscript. FY and AL contributed with ideas and proofread the manuscript. IJ has formulated and supervised the work, and edited the manuscript. All authors have read and approved the final manuscript.

## Figures and Tables

**Table 1:** Extent of potential cross-hybridizations in DNA microarray probes.

Clone set	DB	**Type A (%)**				**Type B (%)**			CHS(A,B)
		= 0	= 1	≥ 2	≥ 2*	= 0	= 1	≥ 2	

Rn 14	RefSeq	63.3	30.7	6.0	16.3	91.3	6.6	1.7	70/200,25
Rn 14	TIGR RGI	5.2	74.6	20.2	21.3	87.7	8.5	3.8	70/200,25
Rn 14	UniGeneUnique	15.9	63.0	21.2	25.2	84.7	11.1	4.2	70/200,25
Rn 14	BeGIn	26.2	62.8	11.1	15.0	82.3	15.4	2.3	70/200,25
Mm 15	RefSeq	34.3	43.2	22.5	34.2	84.5	5.8	9.7	70/200,25
Mm 15	TIGR MGI	47.3	21.5	31.3	59.0	79.5	8.5	12.1	70/200,25
Mm 15	UniGeneUnique	9.9	49.1	40.1	45.0	76.2	11.6	12.3	70/200,25
Mm 15	BeGIn	5.3	57.0	37.8	40.0	78.7	11.5	9.8	70/200,25
Hs 40	RefSeq	13.1	59.5	27.4	31.5	93.3	4.0	2.7	70/200,25
Hs 40	TIGR HGI	1.0	30.8	68.2	68.9	72.5	18.2	9.3	70/200,25
Hs 40	UniGeneUnique	10.9	60.7	28.4	31.8	87.4	7.7	4.9	70/200,25
Mm Oligo	RefSeq	32.5	64.4	3.2	4.7	97.7	1.9	0.3	70/69,20
Mm Oligo	TIGR MGI	59.6	26.2	14.2	35.1	95.8	3.2	1.0	70/69,20
Mm Oligo	UniGeneUnique	9.6	77.7	12.7	14.0	94.8	4.3	0.9	70/69,20
Mm Oligo	BeGIn	15.6	77.1	7.3	8.6	95.7	3.6	0.6	70/69,20

Three cDNA probe sets, human (Hs 40), rat (Rn 14) and mouse (Mm 15), and one mouse oligonucleotide set (Mm Oligo) were checked against cDNA databases (DB). In most cases, a single hit shows that the probe is represented in the DB. Two or more hits indicate potential cross-hybridization. If a hit qualifies as a *type A *hit, it is not considered to be *type B*, even if it satisfies the criteria. The percentages represent the number of probes having the given number of hits, relative to the total number of probes in the probe set. The column ≥ 2* shows the number of probes with two or more hits, relative to the total number of the probes that were found in the database. Default BLAST settings were used, except for word-size (9), and mismatch penalty (-1). The column CHS(A,B) shows the Cross-Hybridization Settings used for *type A *and *type B *search. Total number of probes: Hs 40: 40000, Rn 14:13447, Mm 15:13797 and Mm Oligo:16463.
